# Does the use of different scaffolds have an impact on the therapeutic efficacy of regenerative endodontic procedures? A systematic evaluation and meta-analysis

**DOI:** 10.1186/s12903-024-04064-5

**Published:** 2024-03-09

**Authors:** Feng Yang, Kai Sheng, Lintong Yu, Jun Wang

**Affiliations:** Department of Pediatric Dentistry, Shanghai Ninth People’s Hospital, Shanghai Jiao Tong University School of Medicine; College of Stomatology, Shanghai Jiao Tong University; National Center for Stomatology; National Clinical Research Center for Oral Diseases; Shanghai Key Laboratory of Stomatology; Shanghai Research Institute of Stomatology, Shanghai, 200011 China

**Keywords:** Regenerative endodontic procedures, Pulp regeneration, Pulp necrosis, Immature permanent tooth

## Abstract

**Background:**

In the regenerative endodontic procedures, scaffolds could influence the prognosis of affected teeth. Currently, there is controversy regarding the postoperative evaluation of various scaffolds for pulp regeneration. The objective of this study was to access whether other scaffolds, used alone or in combination with blood clot (BC), are more effective than BC in regenerative endodontic procedures.

**Methods:**

We systematically search the PubMed, the Cochrane Central Register of Controlled Trials (CENTRAL), Embase, and Google Scholar databases. Randomized controlled trials examining the use of BC and other scaffold materials in the regenerative endodontic procedures were included. A random effects model was used for the meta-analysis. The GRADE method was used to determine the quality of the evidence.

**Results:**

We screened 168 RCTs related to young permanent tooth pulp necrosis through electronic and manual retrieval. A total of 28 RCTs were related to regenerative endodontic procedures. Ultimately, 12 articles met the inclusion criteria and were included in the relevant meta-analysis. Only 2 studies were assessed to have a low risk of bias. High quality evidence indicated that there was no statistically significant difference in the success rate between the two groups (RR=0.99, 95% CI=0.96 to 1.03; 434 participants, 12 studies); low-quality evidence indicated that there was no statistically significant difference in the increase in root length or root canal wall thickness between the two groups. Medium quality evidence indicated that there was no statistically significant difference in pulp vitality testing between the two groups.

**Conclusions:**

For clinical regenerative endodontic procedures, the most commonly used scaffolds include BC, PRP, and PRF. All the different scaffolds had fairly high clinical success rates, and the difference was not significant. For regenerative endodontic procedures involving young permanent teeth with pulp necrosis, clinical practitioners could choose a reasonable scaffold considering the conditions of the equipment and patients.

**Supplementary Information:**

The online version contains supplementary material available at 10.1186/s12903-024-04064-5.

## Introduction

Regenerative endodontic procedures (REPs) involve combining scaffolds, stem cells, and signalling factors and then implanting them into the pulp cavity of an affected tooth to replace damaged pulp tissue via tissue engineering [1, 2]. This process promotes the regeneration of blood vessels and nerves in the root canal system and restores the original function of the pulp-dentin complex. Postoperative evaluation of the results of pulp regeneration can be divided into three goals [3, 4]. The first goal is the absence of clinical symptoms and bone tissue healing; the second is an increase in root canal length and root canal wall thickness (desired but not necessary); and the third is a positive pulp vitality test result.

The three key elements of REPs are stem cells, scaffolds and signalling molecules. The ideal scaffold material should have the following characteristics: the ability to provide biological and mechanical support for stem cells, i.e., an environment conducive to cell adhesion, migration, proliferation and differentiation; the ability to facilitate the transportation of nutrients, oxygen and metabolites; and a degradation rate consistent with tissue regeneration. Furthermore, scaffolds are better able to evoke a small inflammatory reaction than other materials and are easy to prepare [5–7]. Scaffolds in common clinical practice include blood clots (BCs), platelet-rich plasma (PRP), platelet-rich fibrin (PRF), and hyaluronic acid.

Different scaffold materials have their own advantages and disadvantages. The BC is the most traditional and most popular type of material used in REPs. It was reported to be related to a high success rate, simplicity, economy, and lack of allergic reactions [8]. However, a BC might not induce true pulp-dentin complex regeneration. The mechanical structure of a BC is relatively fragile and may not be able to fill the root canal during treatment, eventually leading to coronal sealing collapse [9]. PRP is a first-generation autologous platelet concentrate (APC) rich in growth factors obtained by centrifugation of autologous whole blood. Platelets in PRP release important growth factors, including vascular endothelial growth factor (VEGF), platelet-derived growth factor (PDGF), fibroblast growth factor (FGF), and epidermal growth factor (EGF) [10]. Several research results suggest that PRP induces regeneration of periodontal tissue rather than dental pulp tissue [10, 11].

Currently, there is still controversy regarding the postoperative evaluation of various scaffolds for pulp regeneration. For example, several scholars have observed good effects on tooth root growth and canal wall thickening after using BCs alone, or PRP or PRF alone [12, 13]. The expected effect of tooth root growth and thickening accouts for the majority of cases [14–16]; however, according to Bezgin et al. [17], root growth and thickening are observed after using BCs but not after using PRP alone. Several studies have reported that a BC in combination with PRP is effective as a dental pulp regeneration scaffold [18]. Therefore, this meta-analysis aimed to access whether other scaffolds, used alone or in combination with BC, are more effective than BC in regenerative endodontic procedures and to provide a reference for clinical scaffold selection.

## Materials and methods

### Protocol and methods

This systematic review was prospectively registered on the International Prospective Register of Systematic Reviews (INPLASY, INPLASY202410072) and written in accordance with the Preferred Reporting Items for Systematic Reviews and Meta-analyses (PRISMA) guidelines [19].

### Search strategy

We searched the PubMed, the Cochrane Central Register of Controlled Trials (CENTRAL), Embase and Google Scholar databased to identify potentially eligible articles. We developed a search strategy based on PubMed without any language or time restrictions, and the search strategy was applicable to other databases (see Additional file 1). In addition, we searched the reference lists of eligible trials, as well as relevant systematic and narrative reviews. We conducted a manual search of 10 dental-related journals. All the electronic searches and manual searches were last updated in March and February 2023, respectively.

### Eligibility criteria

Two reviewers (F.Y. and T.Y.) screened the titles and abstracts of all retrieved records in duplicate and independently according to the Population, Intervention, Comparison, Outcomes, and Study Design (PICOS) framework. All disagreements were successfully resolved through discussions with two experts (J.W. and K.S.).

- Population (P): Patients with pulp necrosis of young permanent teeth;
- Intervention (I): The use of exogenous scaffolds (alone or combined with BC) for regenerative endodontic procedures;
- Control (C): Self-applied BC alone for regenerative endodontic procedures;
- Outcome measures (O):

The primary outcomes of this review were as follows [20]:

1. Overall success rate
2. Increase in tooth root length
3. Increase in the root canal wall thickness

The secondary outcomes were the results of the pulp vitality test, apical foramen closure and calcification in the root canal after surgery.

- e. Study design (S): Randomized controlled trials (RCTs) with at least 6 months of follow-up.

### Data extraction

Two reviewers (F.Y. and T.Y.) independently extracted relevant data using homemade forms. Any disagreements were resolved by discussion, and a third reviewer was consulted when necessary. We contacted the first or corresponding author of the included studies to obtain missing information. For each trial, the extracted data consisted of five items: general information, study characteristics, patient characteristics, interventions, outcome measurements, and results.

- (1) General information: title, year of publication, country where the study was conducted, journal information and author information.
- (2) Study characteristics: sample size, study date and duration, random allocation method, allocation concealment, and blinding.
- (3) Patient characteristics: age range, sex, tooth position, aetiology of pulp necrosis, type of scaffold used, number of patients, and number of teeth.
- (4) Intervention: intervention type and control type, included BC, PRP, PRF, concentrated growth factor (CGF), platelet pellet (PP), BioGuide membrane, and fibroblast growth factor (FGF).
- (5) Outcome: a detailed description of the outcomes of interest, such as pulp response, periapical healing, root lengthening, canal wall thickening, and apical closure.
- (6) Results: Extract relevant continuous variables and binary variables.

### Data synthesis

We analysed risk ratios (RRs) and mean differences (MDs) for dichotomous data and continuous data, respectively, together with their corresponding 95% confidence intervals (CIs) [21, 22]. Data synthesis was performed using Review Manager software (RevMan 5.4. Copenhagen: The Nordic Cochrane Centre, The Cochrane Collaboration). Pooled data were analysed using a random-effects model, as the CI of the mean effect sizes was wider than that obtained from the fixed-effects model, thus enabling a more conservative interpretation [23].

### Risk of bias (ROB) assessment

The Cochrane risk of bias tool (V 1.0) was used to assess the ROB of the included studies [24]. The tool addresses seven key domains: sequence generation, allocation concealment, blinding of participants and personnel, blinding of assessment, incomplete outcome data, selective reporting, and other biases. Two reviewers (F.Y. and T.Y.) independently performed assessments of all the included studies, and each domain was assessed and given a risk of bias rating of “high,” “low,” or “unclear.” All discrepancies were resolved by discussion with two experts (J.W. and K.S.).

### Sensitivity analysis

For the main meta-analyses of the increase in root length and canal wall thickness, we proposed two forms of sensitivity analysis: removing studies with the shortest observational follow-up period (12 months or less) and removing studies classified as missing standard deviations. We conducted these meta-analyses using a random effects model.

### Assessment of publication bias

When at least 10 studies are included in the meta-analysis, publication bias can be assessed by funnel plots and Egger’s test [24].

### Certainty of evidence

The Grading of Recommendations, Assessment, Development and Evaluation (GRADE) framework was used to assess the certainty of evidence for each primary outcome [21, 22]. The six GRADE criteria were as follows: study design, risk of bias, precision, consistency, publication bias and other considerations. RCTs started with high certainty evidence. Five factors (risk of bias, inconsistency, indirectness, imprecision and publication bias) could downgrade the certainty of the evidence. Based on these criteria, we graded each outcome into four levels of supporting evidence (high, moderate, low, or very low).

## Results

### Literature selection

**Fig. 1:**
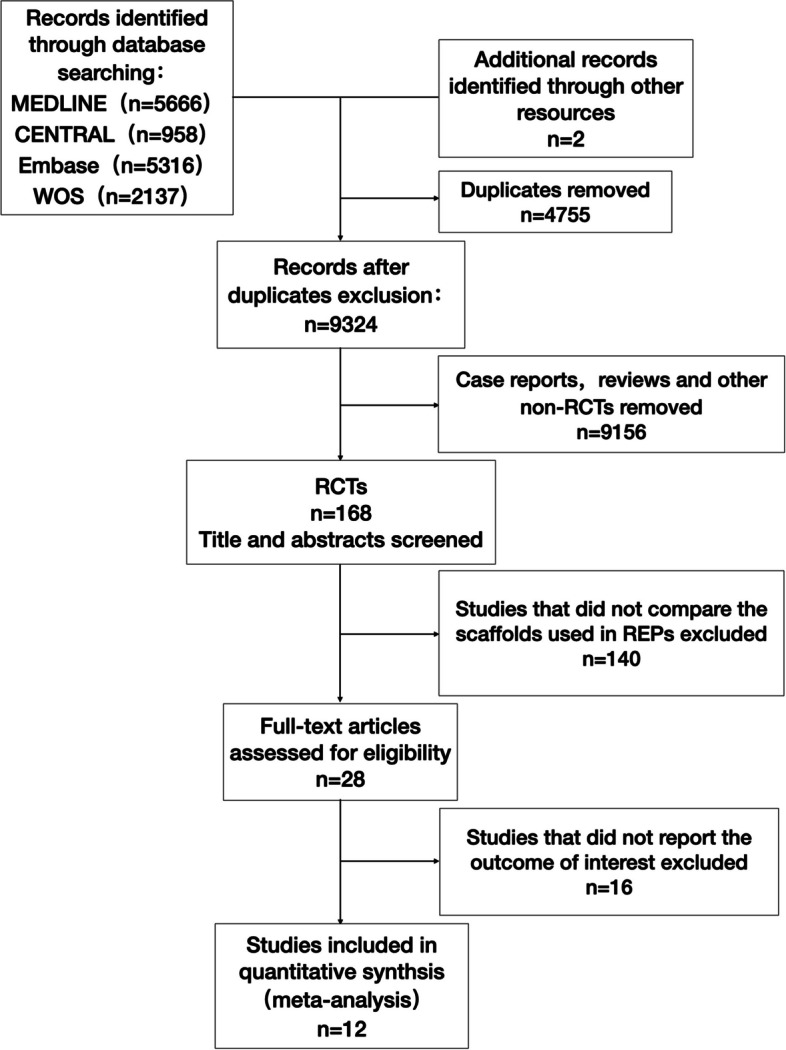
Flow diagram of study selection

We searched the PubMed, CENTRAL, Embase and Google Scholar databases and obtained 5666, 968, 5316 and 2137 records, respectively. Two supplemental records were obtained through manual searching. After removing duplicates, there were a total of 9324 articles related to the treatment of pulp necrosis in young permanent teeth remained (see Additional file 2). Among them, 168 RCTs were screened out, and we reviewed the titles and abstracts. Twenty-eight of these RCTs involved REPs. After reading the full texts, we excluded 16 articles because of the absence of relevant outcomes; ultimately, 12 articles met the inclusion criteria and were included in this meta-analysis (Fig. 1).

### Characteristics of the included studies

#### Trial design and participants

All 12 included RCTs were published between 2012 and 2022, of which 9 were published in the past 5 years. These studies were conducted in the following countries: China, Turkey, Egypt, India, and Saudi Arabia. A total of 407 people participated in these RCTs, which included 481 teeth. The participants in 10 studies were generally between 6 and 14 years old [17, 25–33], while in the other two studies [18, 34], some participants were older, the oldest of which was 28 years in age. The aetiology of necrosis in young permanent teeth mainly include dental trauma [17, 25–30, 32–34], caries [17, 30, 34] and pulp exposure after central cusp fracture [32, 33]. Two articles did not provide the specific aetiology [18, 31].

#### Intervention and comparison

The characteristics and treatment of the included studies are detailed in Table 1. In 12 RCTs, the control group included REPs with BCs. The exogenous scaffolds in the experimental group included PRP, PRF, PP, the Bio-Gide membrane and FGF, with 7 studies reporting on PRP [17, 18, 25, 27, 28, 30, 34] and 4 studies reporting on PRF [26, 27, 29, 34]. Regarding the use of exogenous scaffolds, seven studies used them alone [17, 25, 27–30, 34], while 5 studies used them in combination with BCs [18, 26, 31–33].

**Table 1:** Data extracted from the included studies

SN	Author/year/ country	Age group	AEtiology of pulp necrosis	Sample size recruited	Sample for final follow-up analysis	Follow-up protocol	Clinical success(%)
1	Elsheshtawy et al./2020/ India	Average age 12.66±4.47 y	29 trauma 1 dysplasia (dens invagination)[ in PRP group] 1 unknown ^a^	26 subjects 31 teeth BC-17 vs PRP-14	31 teeth BC-17 vs PRP-14	3, 6, 9, 12 mo	BC-88.2% PRP-85.7%
2	Ragab et al./2019/ Egypt	7-12 y	Trauma	22 subjects 22 teeth BC-11 vs BC+PRF-11	22 teeth BC-11 vs BC+PRF-11	6, 12 mo	BC-100% BC+PRF-100%
3	Rizk et al./2019/ Egypt	8-14 y	Trauma	15 subjects 30 teeth PRP-15 vs BC-15	26 teeth PRP-13 vs BC-13	3, 6, 9, 12 mo	PRP-100% BC-100%
4	Ulusoy et al./2019/ Turkey	8-11 y	Trauma	77 subjects 88 teeth PRP-22 vs PRF-22 vs PP-22 vs BC-22	73 teeth PRP-18 vs PRF-17 vs PP-17 vs BC-21	3, 6, 9, 12mo, thereafter annually (average 28.25±1.20 mo)	PRP-100% PRF-94.2% PP-100% BC-95.3%
5	Shivashankar Et al./2017/ India	6-28 y	Trauma and/ or caries	60 subjects 60 teeth PRF-20 vs BC-20 vs PRP-20	54 teeth PRF-20 vs BC-15 vs PRP-19	3, 6, 9, 12 mo	PRF-90% BC-100% PRP-100%
6	Rizk et al./2020/ Egypt	8-14 y	Trauma	13 subjects 26 teeth PRF-13 vs BC-13	24 teeth PRF-12 vs BC-12	3, 6, 9, 12 mo	PRF-100% BC-100%
7	Alagl et al./2017/ Arabia	8-11 y	24 trauma 6 caries	16 subjects 32 teeth BC-16 vs PRP-16	30 teeth BC-15 vs PRP-15	3, 6, 9, 12 mo	BC-100% PRP-100%
8	Bezgin et al./2015/ Turkey	7-13 y	14 trauma 6 caries	11 subjects 22 teeth BC-11 vs PRP-11	20 teeth BC-10 vs PRP-11	3, 6, 9, 12, 15, 18 mo	BC-100% PRP-100%
9	Nagy et al./2014/ Egypt	9-13 y	Unknown	24 subjects 24 teeth BC-12 vs BC+bFGF-12	20 teeth BC-10 vs BC+bFGF-10	3, 6, 12, 18 mo	BC-90% BC+bFGF-80%
10	Jiang et al./2017/ China	Average of the control group 9.82±1.5 y Average of experimental group 10.3±1.9 y	14 trauma 29 broken central cusp	43 subjects 46 subjects BC-23 vs BC+Bio-Gide-23	43 teeth BC-22 vs BC+Bio-Gide-21	Every 3 mo, at least 6 mo	BC-100% BC+Bio-Gide-100%
11	Jiang et al./2022/ China	Average of the control group 10.6 ± 1.7 y Average of experimental group 11.0 ± 1.9 y	21 trauma 55 broken central cusp	80 subjects 80 teeth BC-40 vs BC+Bio-Gide-40	76 teeth BC-38 vs BC+Bio-Gide-38	Every 3 mo, at least 6 mo	BC-100% BC+Bio-Gide-100%
12	Jadhav et al./2012/ India	15-28 y	Unknown	20 subjects 20 teeth BC+PRP-10 vs BC-10	20 teeth BC+PRP-10 vs BC-10	6, 12 mo	BC+PRP-100% BC-100%

^a^The aetiology of the one tooth was unspecified in the text

#### Outcome measures

All 12 RCTs reported relevant outcomes at 6 and 12 months, with the longest average follow-up time being 28 months [27]. All of those studies reported clinical success rates; six studies [26–28, 31–33] reported an increase in root length and five studies [27, 28, 31–33] reported an increase in root canal wall thickness. The outcomes of increments in length and thickness were reported as the percentage increase in root length or width. Six studies reported the results of pulp vitality testing. The method involved cold tests combined with electrical vitality tests [17, 27, 30]; two studies used only an electrical vitality test [32, 33], and one did not state the method used [34]. Four studies reported adverse outcomes, including postoperative intracanal calcification [17, 32, 33], crown discolouration [17, 29, 32, 33], and coronal mineral trioxide aggregate (MTA) collapse [33].

#### Risk of bias (ROB) assessment

**Fig. 2:**
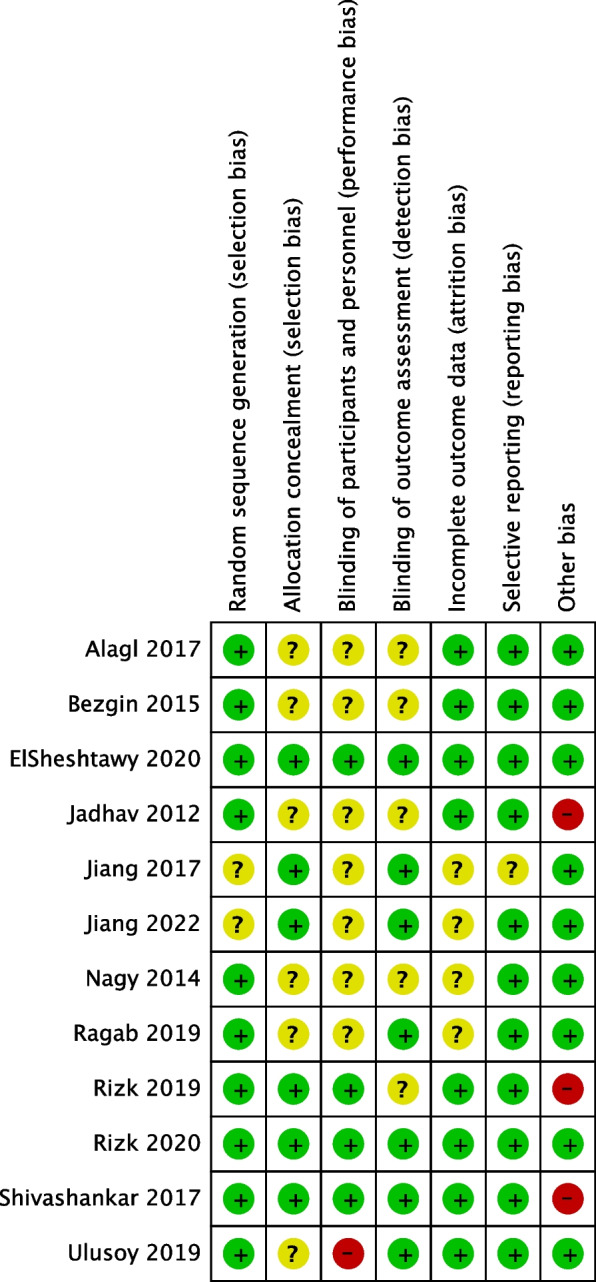
Risk of bias summary graph: review authors’ judgement regarding the risk of bias for the included studies

The risk of bias in the 12 included studies is shown in Fig. 2. We successfully obtained all the full texts and therefore were able to assess the risk of bias for all trials included in this meta-analysis. According to the assessments of two reviewers (F. Y. and T. Y.), good consistency was achieved in 7 domains of the 12 studies. Only 2 studies were assessed to be at low risk in all domains and could be judged to have a low risk of bias [25, 29]. According to the overall assessment of risk of bias, all 12 studies exhibited good reporting bias.

**Fig. 3:**
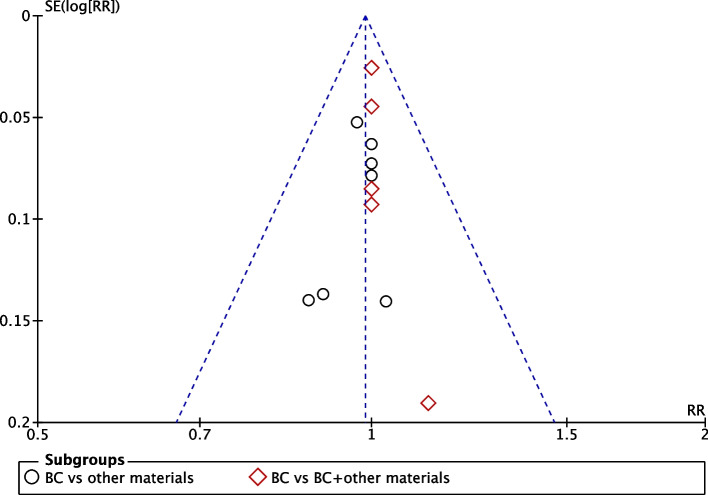
Funnel plot of reporting bias in the clinical success rate

We also evaluated publication bias, and the funnel plot of the clinical success rate showed no reporting bias (Fig. 3). Based on the GRADE assessment, the certainty of the evidence for the meta-analysis using clinical success rates was assessed as high certainty. The certainty of the evidence for the meta-analysis using pulp viability testing and postoperative intracanal calcification was assessed as moderate certainty, while that using the increment of root length and wall thickness was assessed as low certainty (see Table 2).

**Table 2:** Certainty of the evidence (GRADE) for the meta-analysis using the clinical success rate, increment in root length, increment in root canal wall thickness, pulp vitality test, and postoperative intracanal calcification

Outcomes	Included studies(participants)	Type of study	Factors that downgrade the certainty of the evidence					Relative effect (95% CI)	Certainty of the evidence
			Risk of bias	Indirectness	Inconsistency	Imprecision	Publication bias		
Clinical success rate	12 (240)	RCT	Not downgraded	Not downgraded	Not downgraded	Not downgraded	Not downgraded	RR 0.99 (0.94,1.05)	⊕⊕⊕⊕/A
Increment of root length	6 (145)		Not downgraded	Not downgraded	Downgraded^a^	Downgraded ^2^	/	MD -1.02 (-4.94,2.91)	⊕⊕⊝⊝/C
Increment of root canal wall thickness	5 (134)		Not downgraded	Not downgraded	Downgraded^a^	Downgraded ^2^	/	MD -5.09 (-11.46,1.28)	⊕⊕⊝⊝/C
Pulp vitality test	6 (175)		Not downgraded	Not downgraded	Downgraded^a^	Not downgraded	/	RR 0.77 (0.61,0.97)	⊕⊕⊕⊝/B
Postoperative intracanal calcification	2 (59)		Not downgraded	Not downgraded	Not downgraded	Downgraded^2^	/	RR 1.12 (0.80,1.58)	⊕⊕⊕⊝/B

^a^Refers to a significant inconsistency (I^2^ > 80% and *P* < 0.1); 2 refers to a sample size that was not sufficient, which led to a overly wide confidence interval; A refers to a high certainty of evidence, B refers to a moderate certainty of evidence and C refers to a low certainty of evidence

#### Effects of interventions

The effect of different scaffolds on REPs for pulp necrosis in young permanent teeth was reported in various ways for all the included studies. The following results are separately reported in this systematic review:

(1) Clinical success rate, (2) increase in root length, (3) increase in root canal wall thickness, (4) pulp vitality test results, and (5) other results.

#### Clinical success rate

**Fig. 4:**
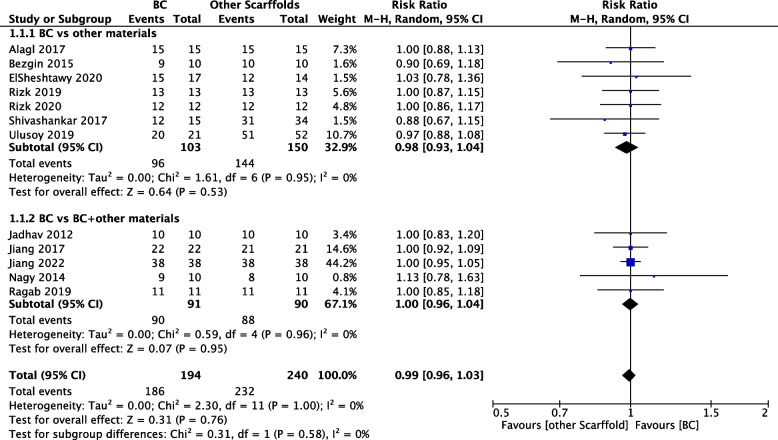
Comparison of other scaffolds versus BCs by outcome: clinical success rates

A total of 12 studies were included in the clinical success rate comparison between the BC group and the other scaffolds group. As shown in Fig. 4, the pooled RR (random-effects meta-analysis) of the clinical success rate was 0.99 (95% CI=0.96 to 1.03; 434 participants, 12 studies), which suggested a nonsignificant effect. The heterogeneity of the outcome was not statistically significant (χ^2^=2.30,df=11, *P*=1.00, I^2^=0%).

#### Increment of tooth root length

**Fig. 5:**
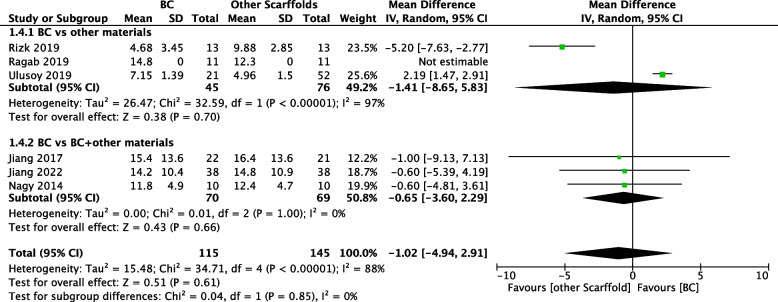
Comparison of other scaffolds versus BCs by outcome: Increment of tooth root length

According to the random effects models, the pooled estimate for the increase in tooth root length was -1.02%, as shown in Fig. 5 (95% CI: -4.94,2.91; 260 participants; 6 studies [26–28, 31–33]), which suggested a nonsignificant effect in favour of the use of other exogenous scaffolds. The heterogeneity of the outcome was significant (χ^2^=34.71, df=4, *P*<0.00001, I^2^=88%); therefore, we conducted a subgroup analysis based on whether the exogenous scaffold was used alone or combined with BCs. We found that the outcomes of the two studies had opposite tendencies with increasing root length, resulting in considerable heterogeneity [27, 28].

#### Increase in root canal wall thickness

**Fig. 6:**
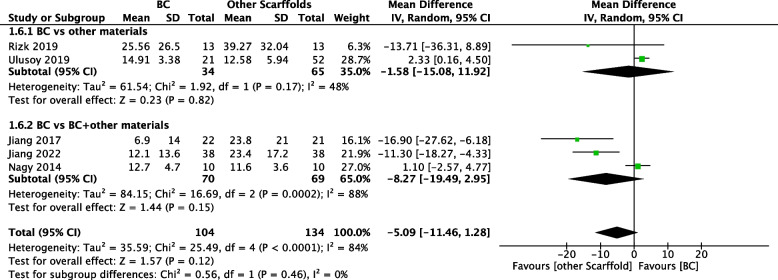
Comparison of other scaffolds versus BC by outcome: Increase in root canal wall thickness

The exogenous scaffold group performed better in terms of the increment of root canal wall thickness, but the difference between the two groups was not statistically significant (MD=-5.09%; 95% CI=-11.46 to 1.28; 238 participants, 5 studies [27, 28, 31–33]). Similarly, due to the different tendencies of the results of the three studies [31–33], there was significant heterogeneity (χ^2^=25.49, df=4, *P*<0.00001, I^2^=84%) (see Fig. 6).

#### Pulp vitality test results

**Fig. 7:**
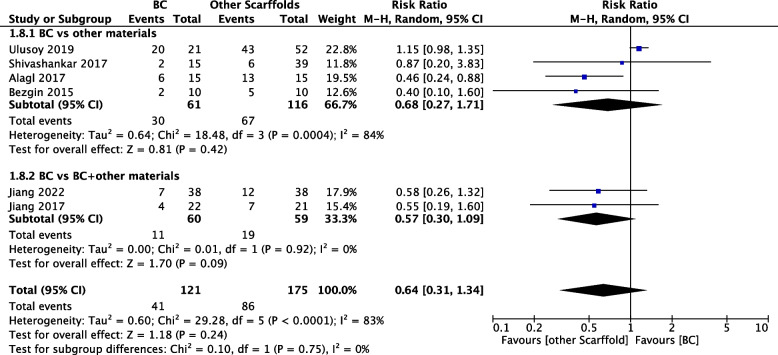
Comparison of other scaffolds versus BCs by outcome: Pulp vitality test results

As shown in Fig. 7, the pooled RR (random-effects meta-analysis) of postoperative pulp vitality test results was 0.64 (95% CI 0.31 to 1.34; 296 participants, 6 studies [17, 27, 30, 32–34]), which suggested a nonsignificant effect in slightly favouring the use of other exogenous scaffolds. The results were highly heterogeneous (χ^2^=29.28, df=4, *P*<0.00001, I^2^=83%).

#### Other outcome measures

The outcomes of the meta-analysis of apical foramen closure and root canal calcification are separately shown in Electronic Appendix Figs. 1 and 2, respectively. Moreover, there was no statistically significant difference between the exogenous scaffold groups and the BC groups.

#### Subgroup analyses

We performed subgroup analyses on the use of exogenous scaffolds alone or in combination with BCs; the results are shown in Figs. 4, 5, 6 and 7. We found that there was no significant difference in the clinical success rate, increase in root length, increase in root wall thickness or pulp vitality test results among the subgroups. Similarly, the outcome of subgroup analyses of apical foramen closure and root canal calcification suggested a nonsignificant difference (see Electronic Appendix Figs. 1 and 2). In addition, we assumed that the aetiology of dental pulp necrosis also affects the prognosis. Based on these limited data, we conducted a subgroup analysis of the clinical success rate in studies where the cause was only dental trauma and studies where the cause was not limited to dental trauma. The subgroup analysis showed no statistically significant difference (see Electronic Appendix Fig. 3).

## Discussion

In our systematic review, the clinical and imaging results of the BC group and other exogenous scaffold groups were comparable, and the overall success rates were both greater than 90%, similar to the rates in previous clinical trials [35, 36]. Notably, the success criteria for pulp regeneration may differ across clinical trials. According to the American Association of Endodontists (AAE) clinical guidelines, the success criteria include the disappearance of clinical symptoms and apical radiolucency, further root development, and a positive pulp vitality test [4]. In the included studies, for example, Ulusoy et al. divided the judgement criteria into three levels: failure; only the absence of clinical symptoms along with radiographic evidence of osseous healing; radiographic root development and a positive pulp vitality test result [27]. The latter two levels were both categorized as success, which was not consistent with the AAE criteria. In addition, some scholars believe that crown discolouration and root canal calcification are inevitable in REPs and should not be considered a failure [13]. Others believe that preventing tooth discolouration, especially in the aesthetic area, should also be included in the success criteria [35]. When using BCs, the unpredictability of blood clotting increases the likelihood of tooth discolouration [37]. Unclear or varying success criteria for the included trials may bias the final analysis of the results.

This meta-analysis revealed that there was no statistically significant difference in the effects of BCs or other exogenous scaffolds on the further development of tooth roots. Some scholars believe that residual bacteria after REPs affect the development of dentine root canal wall thickness [37, 38]. Conventional BC methods involve limited antibacterial media, but in theory, APCs contain a high concentration of growth factors, which promote stem cell migration, proliferation and differentiation, as well as strong and stable fibrous matrix and antibacterial properties. Similarly, in the included trials, Rizk et al. hypothesized that the root development of the PRF group would be better than that of the BC group [29]. In addition to the above reasons, the author's explanation is that the thrombin contained in PRF can create equal-sided junctions in polymerized fibrin so that signalling molecules can discharge continuously and the fibrin network is mouldable, ultimately forming a proper microenvironment for cell migration. Notably, according to the clinical guidelines of the AAE, an increase in root canal wall thickness is usually observed 12-24 months after treatment [4]. Nevertheless, we observed that the maximum follow-up period of some included studies was only 12 months, and some of them were analysed in forest plots regarding root development [28, 32]. Therefore, the impact of differences in follow-up time on the final results was also not meausrable. In addition, we noticed that for the calculation of imaging data, many experiments did not consider the impact of changes in camera angles into consideration, and their methods of correction were also different, making it impossible to ensure the comparability of data between groups.

The AAE proposes that a positive pulp vitality test (cold test or electric vitality test) is the highest goal of REPs [4]. However, the results of the pulp vitality test in the included trials varied greatly, and in some studies, neither the control group nor the experimental group exhibited a positive result. Therefore, we selected only studies with discrepant data for the two groups for meta-analysis. Electric vitality testing may result in false negatives for young permanent teeth with an open apical foramen, which means that the true result was based on the closure of the apical foramen. Moreover, when cold testing is used, as the pulp-capping material is often placed slightly below the plane of the cemento-enamel junction, vital pulp regeneration will not occur in the crown region. This means that when cold stimuli are applied to a crown, they cannot be transferred to the tissue under the capping material, which also leads to false negatives [34].

The adverse outcome reports of REPs have not been widely been considered. At present, the most common complication is calcification in the root canal, for which the incidence rate is approximately 50% [17, 32, 33]. In a previous study by Chen et al., the incidence rate was similar to that of 35% [39]. Biological analysis of the causes of root canal calcification has shown that blood from the apical foramen may bring periodontal stem cells and alveolar bone-derived bone marrow stem cells, ultimately inducing the formation of bone or cementum structures in the root canal [40–42]. In addition, some studies have shown that residual plaque biofilms and antigens are related to root canal calcification, and that in the presence of both, stem cells in the apical papilla stably express osteoblast-like markers [43]. Currently, studies on the long-term prognosis of patients with root canal calcification after REPs are rare. Therefore, whether calcification must be avoided is yet unclear. We suggest taking calcification into consideration when choosing the scaffold, as root canal calcification, especially obliteration, is widely accepted to be detrimental when root canal treatment is needed.

Regarding the ROB assessment, double-blinding of patients and personnel may have been impossible because the APC group needed to undergo the treatment process of blood drawing treatment process, and the patients would have realized that they were assigned to the APC group [27]. Rizk solved this problem through the design of "a split mouth" to ensure that each participant needed to draw blood, and the two affected teeth of a participant underwent different groups of operations [28, 29]. Whether double-blinding affects the results of REPs cannot be determined, and we recommend that researchers conducting subsequent trials take note of this.

Among the current studies screened, there was only one network meta-analysis focused on the comparison of exogenous scaffolds and traditional BC methods in dental pulp regeneration, but it included many non-RCTs, which decreased the certainty of the evidence [44]. In contrast, this review included only RCT studies, excluding the possibility that cases using exogenous scaffolds were due to poorer tooth conditions or unsuccessful bleeding, thus ensuring the validity of the results and conclusions.

This review also has certain limitations. Due to the different study designs, data standards, data integrations and outcome measurement methods of the included trials, comparison of teeth root development between BCs and other scaffolds have been limited. In addition, we simply categorized all the exogenous scaffolds into groups for comparison with BC, which was due to the limited number of studies available. This could have resulted in significant heterogeneity. At present, more clinical trials are still needed to verify the effect of exogenous scaffolds compared with traditional BC methods.

## Conclusion

Based on the limited evidence of this review, we draw the following conclusions.

For clinical REPs, the most commonly used scaffolds include BC, PRP and PRF. There is high-level evidence that these scaffolds all have high clinical success rates, and the differences are not statistically significant. The methods used to measure the increase in root length and root canal wall thickness, the measurement methods are highly heterogeneous, and based on the currently limited data, there was no significant difference between the use of exogenous scaffolds and traditional BCs, regardless of whether the former was applied alone or in combination with BC, Pulp vitality testing is still not taken seriously by some researchers. Cold testing and electrical vitality testing are recommended methods, but attention should be given to avoid false negatives. For REPs of young permanent teeth with pulp necrosis, clinicians can choose reasonable scaffolds based on the equipment conditions and patient conditions.

### Supplementary Information

**Additional file 1. Appendix Table 1.** Search strategy used for each database.

**Additional file 2: Appendix Figure 1.** Comparison of other scaffolds versus BCs by outcome: Apical foramen closure.

**Additional file 3: Appendix Figure 2.** Comparison of other scaffolds versus BCs by outcome: Root canal calcification.

**Additional file 4: Appendix Figure 3.** Comparison of other scaffolds versus BCs by outcome: Clinical success rate, subgroup analysis of aetiology.

## Data Availability

All data analyzed during this study are included in this manuscript.

## Abbreviations

REPs: regenerative endodontic procedures
BC: blood clot
APC: autologous platelet concentrate
PRP: platelet rich plasma
PRF: platelet rich fibrin
VEGF: vascular endothelial growth factor
PDGF: platelet-derived growth factor
FGF: fibroblast growth factor
EGF: epidermal growth factor
PRISMA: Preferred Reporting Items for Systematic Reviews and Meta-analyses
GRADE: Cochrane Central Register of Controlled Trials
PICOS: Population, Intervention, Comparison, Outcomes, and Study Design
RCT: randomized controlled trial
CGF: concentrated growth factor
PP: platelet pellet
FGF: fibroblast growth factor
RR: risk ratio
MD: mean difference
CI: confidence interval
ROB: risk of bias
GRADE: Grading of Recommendations, Assessment, Development and Evaluation
MTA: mineral trioxide aggregate
AAE: American Association of Endodontists
