# Supplementary figures and images for: Does the use of different scaffolds have an impact on the therapeutic efficacy of regenerative endodontic procedures? A systematic evaluation and meta-analysis
Source: BMC Oral Health. 2024 Mar 9;24:319. doi: 10.1186/s12903-024-04064-5 (PMC10924999; doi:10.1186/s12903-024-04064-5)

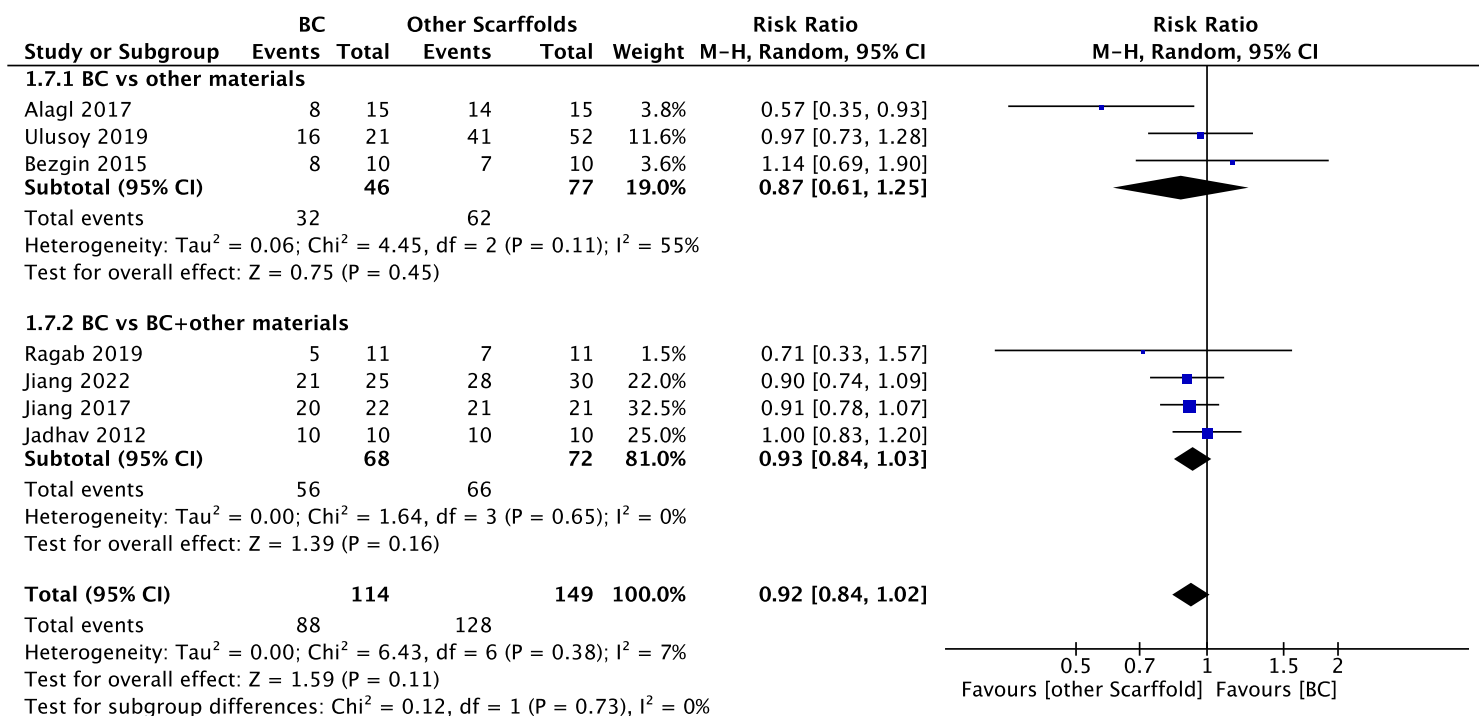

Supplement: Supplementary file 2 — Additional file 2: Appendix Figure 1. Comparison of other scaffolds versus BCs by outcome: Apical foramen closure. [file 12903_2024_4064_MOESM2_ESM.pdf]

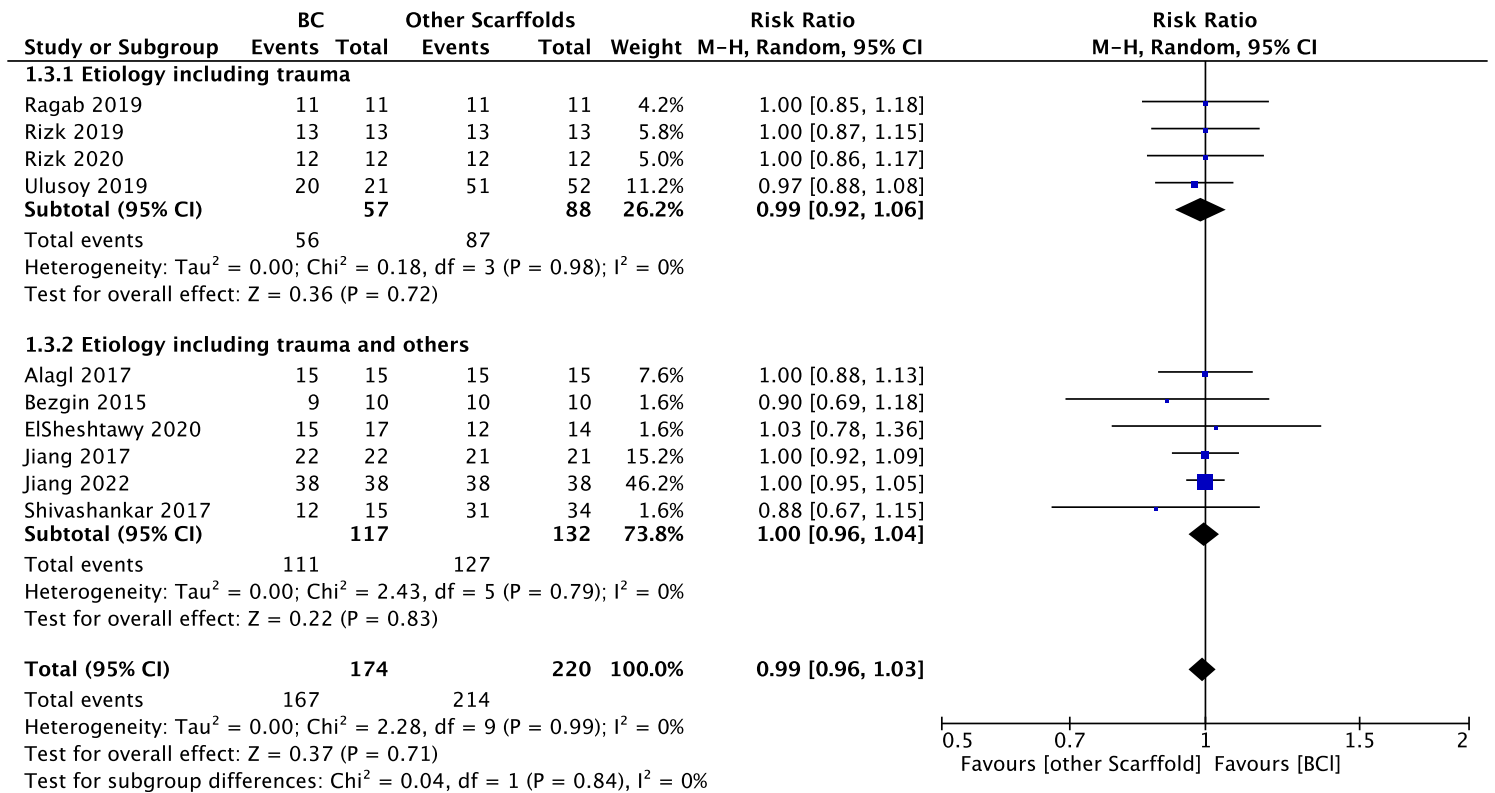

Supplement: Supplementary file 4 — Additional file 4: Appendix Figure 3. Comparison of other scaffolds versus BCs by outcome: Clinical success rate, subgroup analysis of aetiology. [file 12903_2024_4064_MOESM4_ESM.pdf]
