# Supplementary material for: Does the use of different scaffolds have an impact on the therapeutic efficacy of regenerative endodontic procedures? A systematic evaluation and meta-analysis
Source: BMC Oral Health. 2024 Mar 9;24:319. doi: 10.1186/s12903-024-04064-5 (PMC10924999; doi:10.1186/s12903-024-04064-5)

| Study or Subgroup | BC Events | BC Total | Other Scarffolds Events | Other Scarffolds Total | Weight | Risk Ratio M-H, Random, 95% CI |
|---|---|---|---|---|---|---|
| Bezgin 2015 | 4 | 10 | 4 | 11 | 9.7% | 1.10 [0.37, 3.27] |
| Jiang 2017 | 12 | 22 | 10 | 21 | 33.4% | 1.15 [0.64, 2.06] |
| Jiang 2022 | 20 | 38 | 18 | 38 | 56.9% | 1.11 [0.71, 1.74] |
| **Total (95% CI)** | | **70** | | **70** | **100.0%** | **1.12 [0.80, 1.58]** |
| Total events | 36 | | 32 | | | |

Heterogeneity: $Tau^2 = 0.00$; $Chi^2 = 0.01$, df = 2 (P = 1.00); $I^2 = 0\%$
Test for overall effect: Z = 0.66 (P = 0.51)

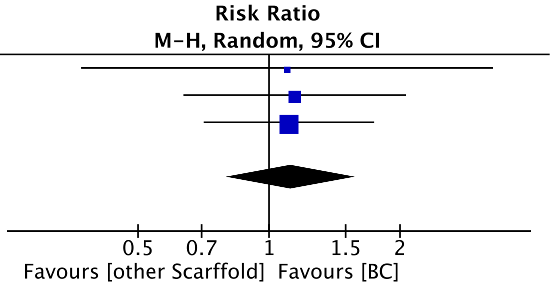

Supplement: Supplementary file 3 — Additional file 3: Appendix Figure 2. Comparison of other scaffolds versus BCs by outcome: Root canal calcification. [file 12903_2024_4064_MOESM3_ESM.pdf]
